# Supplementary material for: Analysis on synergistic cocontraction of extrinsic finger flexors and extensors during flexion movements: A finite element digital human hand model
Source: PLoS One. 2022 May 11;17(5):e0268137. doi: 10.1371/journal.pone.0268137 (PMC9094536; doi:10.1371/journal.pone.0268137)
Supplement: Attachment — Submitted filename: Response to Reviewers.docx [file pone.0268137.s002.docx]

Dear Editors and Reviewers:

Thank you for your letter and for the reviewers’ comments concerning our manuscript entitled “PONE-D-21-23164”. Those comments are all valuable and very helpful for revising and improving our paper, as well as the important guiding significance to our researches. We have studied comments carefully and have made correction which we hope meet with approval. Revised portion were marked in red in the paper. The main corrections in the paper and the responds to the editors and reviewers’ comments were addressed point by point below.

**Responds to the reviewers’ comments:**

**Reviewer #1:**

1. Response to comment: Model inaccuracy: missing muscles and anatomical structures.

Authors responded that the intrinsic hand muscles and extensor mechanism are ‘ignored’ in this model because they are basically ‘difficult to model’. They mentioned one study (Chang et al., 2017) from the applied ergonomics field to provide rationale for excluding intrinsic muscles. However, first, this study was looking at a specific “triggering” motion (concurrent flexion of the DIP and PIP joints without much MCP flexion), which does not require intrinsic action (this is close to intrinsic minus motion). Second, some studies they mentioned (e.g., Valero-Cuevas et al., 1998) did include intrinsic actions, so their answer to this question is basically inaccurate.

More importantly, in my opinion, the authors should focus on providing a proper rationale, i.e., why these muscles or mechanisms were not included in this study (e.g., if their force contribution is negligible) to respond to this reviewer's question. But instead, throughout their response the authors emphasized basically how “difficult” it is to include the intrinsic muscles or the extensor mechanism – the response should be, to properly justify their model selection, why the effects of the intrinsic muscles and/or the extensor mechanism on the model output is small enough (negligible) to exclude these structures. Note that, unfortunately, that will not be the case (i.e., the contribution of intrinsic muscles are quite significant indeed) – see Maier and Hepp-Reymond (1995) and/or Milner and Dhaliwal (2002), which emphasized the importance of intrinsic muscles during force production tasks (similar to what was done in this study).

**Response:**

Thank you for your comments on our article. The effect of the intrinsic muscles and/or the extensor mechanism on the model of this article was indeed insignificant. The literatures suggest that the contribution of the intrinsic muscles of the hand is relevant to the target task. For example, Li et al. (Li Z M , Zatsiorsky V M , Latash M L . Contribution of the extrinsic and intrinsic hand muscles to the moments in finger joints[J]. Clinical Biomechanics, 2000, 15( 3):203-211.) analyzed the contribution of intrinsic and extrinsic muscles to the knuckle moment using the experimental apparatus shown in Figure 1 in conjunction with a biomechanical model of the index finger and concluded: when the point of force application was on the distal phalanx, the force of the INT of the index finger accounted for only 22.5% of the combined FDP and FDS force. The moment contribution of the INT at the MCP joint of the index finger was only 12.4%. In further work, Li et al. (Li Z M , Zatsiorsky V M , Latash M L . The effect of finger extensor mechanism on the flexor force during isometric tasks[J]. Journal of Biomechanics, 2001, 34(8):1097-1102.) stated: when the point of force application was at the distal phalanx, the extrinsic flexor muscles flexor digitorum profundus (FDP) and flexor digitorum superficialis (FDS) accounted for over 80% of the summed force of all flexors, and therefore were the major contributors to the joint flexion at the distal interphalangeal (DIP), proximal interphalangeal (PIP), and metacarpophalangeal (MCP) joints. When the point of force application was at the DIP joint, the FDS accounted for more than 70% of the total force of all flexors, and was the major contributor to the PIP and MCP joint flexion. When the force of application was at the PIP joint, the intrinsic muscle group was the major contributor for MCP flexion, accounting for more than 70% of the combined force of all flexors. The results suggested that the effects of the extensor mechanism on the flexors were relatively small when the location of force application was distal to the PIP joint. When the external force was applied proximally to the PIP joint, the extensor mechanism had large influence on force production of all flexors.

Maier and Hepp-Reymond (M.A. Maier, M.C.Hepp-Reymond. EMG activation patterns during force production in precision grip[J]. Experimental Brain Research, 1995,103:108-122.) reported that the intrinsic muscles of the index finger and thumb were closely related to the low isometric forces generated between the thumb and index finger in the precision grasp experiment (Figure 2).

It can be seen that the contribution of the intrinsic muscles of the hand is related to the target task. The target task (fingertip force) addressed in this paper is very similar to the distal phalanx loading in the work of Li et al. Under this task, the extrinsic muscles are the main contributors to joint flexion of the DIP, PIP and MCP joints (accounting for more than 80% of the total force of all flexors). Therefore, the simplification of the intrinsic muscles and the extensor mechanism in the modeling of this paper is justified.

Admittedly, the intrinsic muscles are important components of the hand muscles, and a whole-hand model containing the intrinsic muscles has still not been created. The modeling and loading of intrinsic muscles in the whole hand model is worthy of consideration and is something we intend to carry out in further work (page 18, lines 308-315).

2 .Response to comment: Flawed model validation

The authors now provided details of the model parameter estimation process – which is basically estimating material properties of the tendons, ligaments, and joint stiffness. This is a problematic in itself since all these components are connected in series, which means that the effects of these parameters on measured data (fingertip force/MTJ motion) are intertwined (and cannot be told from each other). In other words, given the motion tested (concurrent flexion of all finger joints), there is no way for the model to distinguish the effects of joint stiffness from those of tendon stiffness. Thus, the outcome may appear to make sense, but there is no way to validate the results (or whether any of these estimated parameter values is reliable). Hypothetically, the solutions obtained by the authors may estimate the movement of MTJ correctly, but could lead to 5-fold overestimation of tendon stiffness in combination with 5-fold underestimation of joint stiffness. Therefore, it is possible that this model will work on the dataset collected in this experiment ('interpolation'), but won't be applicable to any other cases ('extrapolation').

**Response:**

The influence of the material parameters to be determined for the model on the measured data can be divided into two cases: the stiffness of tendons and ligaments was positively correlated (to different degrees) with the fingertip force, and the stiffness of the two groups of joints (IP and MCP joints) was negatively correlated (to different degrees) with the fingertip force. All parameters were approximated iteratively using experimental data on the basis of initial values (literature data). And four sets of data were sufficient to determine the four unknowns. The effect of each parameter on the measured data was not "proportional", so that the the solutions for parameter estimation would not lead to "5-fold overestimation of tendon stiffness in combination with 5-fold underestimation of joint stiffness ". Otherwise the determined material parameters would not satisfy the " MTJ-flexion pattern" data in the model validation, even if the four sets of "MTJ-fingertip force" data were satisfied simultaneously. In fact, the model validation process could be regarded as the "extrapolation" of the model after the parameters were determined.
